# Differential effects of tactile high- and low-frequency stimulation on tactile discrimination in human subjects

**DOI:** 10.1186/1471-2202-9-9

**Published:** 2008-01-23

**Authors:** Patrick Ragert, Tobias Kalisch, Barbara Bliem, Stephanie Franzkowiak, Hubert R Dinse

**Affiliations:** 1 Institute for Neuroinformatics, Department of Theoretical Biology, Experimental Neurobiology Lab, Ruhr-University, 44780 Bochum, Germany; 2 Department of Neurology, BG-Kliniken Bergmannsheil, Ruhr-University, 44789 Bochum, Germany; 3 National Institute of Neurological Disorders and Stroke, National Institutes of Health, Human Cortical Physiology Section, Bethesda, MD 20892, USA

## Abstract

**Background:**

Long-term potentiation (LTP) and long-term depression (LTD) play important roles in mediating activity-dependent changes in synaptic transmission and are believed to be crucial mechanisms underlying learning and cortical plasticity. In human subjects, however, the lack of adequate input stimuli for the induction of LTP and LTD makes it difficult to study directly the impact of such protocols on behavior.

**Results:**

Using tactile high- and low-frequency stimulation protocols in humans, we explored the potential of such protocols for the induction of perceptual changes. We delivered tactile high-frequency and low-frequency stimuli (t-HFS, t-LFS) to skin sites of approximately 50 mm^2 ^on the tip of the index finger. As assessed by 2-point discrimination, we demonstrate that 20 minutes of t-HFS improved tactile discrimination, while t-LFS impaired performance. T-HFS-effects were stable for at least 24 hours whereas t-LFS-induced changes recovered faster. While t-HFS changes were spatially very specific with no changes on the neighboring fingers, impaired tactile performance after t-LFS was also observed on the right middle-finger. A central finding was that for both t-LFS and t-HFS perceptual changes were dependent on the size of the stimulated skin area. No changes were observed when the stimulated area was very small (< 1 mm^2^) indicating special requirements for spatial summation.

**Conclusion:**

Our results demonstrate differential effects of such protocols in a frequency specific manner that might be related to LTP- and LTD-like changes in human subjects.

## Background

Since its discovery in the early 1970s, long-term potentiation (LTP) and long-term depression (LTD) of synaptic transmission had been suggested to be crucial factors for activity-dependent changes in the strength of synaptic connections and efficiency of synaptic signal transduction [1,2]. Despite the fact that the outcome of electrical pulse protocols in slice preparations can differ in several brain regions with respect to temporal aspects, duration and intensity of stimulation, some general key properties became apparent: Electrical stimulation using high-frequency bursts of ≥ 5 Hz for several minutes usually results in LTP, which is mediated by glutamatergic synapses, e.g. N-methyl-D-aspartate (NMDA) receptors [3,4]. Unlike LTP, LTD is induced by low-frequency (usually 1–5 Hz) stimulation and results in a suppression of synaptic transmission as marked by a strengthening of inhibitory postsynaptic potentials (IPSPs).

Accordingly, bidirectional synaptic modifications such as LTP and LTD are believed to be crucial mechanisms underlying learning-induced cortical plasticity. However, there is an on-going debate how synaptic plasticity links to systemically observable changes such as cortical map reorganization, and changes in behavior and perception. In human subjects, it is difficult to study the outcome of synaptic modifications on behavioral changes induced by stimuli that drive LTP or LTD-like processes in vivo. Recently, evidence for the functional relevance of such stimulation protocols has been provided in human pain perception. High-frequency stimulation of cutaneous afferents of the proximal forearm resulted in a long-lasting increase in perceived pain to electrical test stimuli. On the other hand, low-frequency stimulation decreased the individual pain perception [5].

In previous studies we took advantage of a stimulation protocol developed in our group that allows for full control of the spatio-temporal pattern of tactile stimuli. In this protocol, we stimulated the tip of the index finger with irregular, Poisson-distributed trains with an average frequency of 1 Hz (minimal 0.3 Hz, maximal 10 Hz), which led to improvement of tactile perceptual performance of the index finger in parallel to expansion of cortical representation of the fingers [6-13]. Plastic changes in primary sensory cortical areas induced by tactile performance changes are typically characterized by substantial spatial selectivity that arises from the presence of well-ordered topographic maps. Accordingly, limited generalization and transfer is taken as evidence for effects in early representations. Peripheral tactile stimulation using irregular pulse trains seems to induce spatially selective changes in performance only on the stimulated index finger rather than a global increase in perceptual performance on non-stimulated fingers of the same hand or the index-finger of the contralateral hand [7].

To evoke plastic processes in the cortical representations of the stimulated skin sites, in the present study we delivered regular trains of either high-frequency (HFS) or low-frequency (LFS) tactile stimuli to the tip of the index finger by means of small stimulation devices (for review see [10]). We hypothesized that HFS leads to an improvement in 2-point discrimination whereas LFS impairs performance.

As an output measure we used tactile spatial 2-point discrimination performance as a marker for its perceptual relevance. Here we demonstrate that brief periods (20 minutes) of intermittent high-frequency (HFS @ 20 Hz) and continuous low-frequency (LFS @ 1 Hz) protocols of tactile stimulation applied to the index-finger (d2) of the right hand evokes significant and long-lasting changes in tactile discrimination behavior.

## Results

### Tactile high-frequency stimulation (t-HFS)

#### Effect of large-field tactile HFS on discrimination thresholds of the right d2

**Figure 1:**
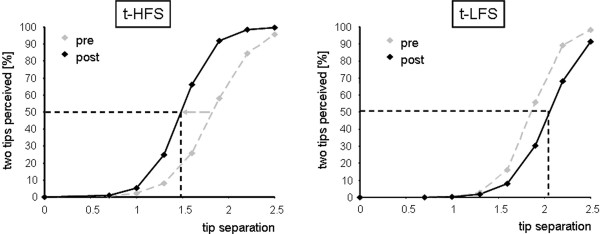
Psychometric functions (regression curves) illustrating the differential effect of 20 min large-field t-HFS and t-LFS stimulation in two representative subjects. Correct responses in percent are plotted as a function of separation distance. 50% level of correct responses is indicated together with resulting thresholds (dashed horizontal and vertical lines). Dashed grey lines show pre-condition before, solid black lines post-condition immediately after t-HFS or t-LFS. After t-HFS there is a distinct shift in the psychometric functions towards lower separation distances (threshold was reduced from 1.83 to 1.48 mm after t-HFS). After t-LFS we found an analogous shift in the psychometric curve, but towards larger separations (threshold was increased from 1.86 to 2.05 mm after t-LFS

**Figure 2:**
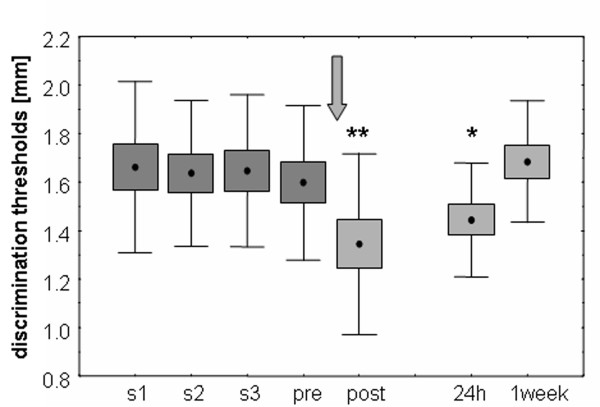
Psychophysical effect of large-field t-HFS on tactile discrimination thresholds of the right d2. Average data from all subjects of group 1 (n = 14). Dots represent mean thresholds, boxes show standard errors, and whiskers correspond to the standard deviation. Time of t-HFS application (20 minutes) on the right d2 is indicated by an arrow. Shown are the results from 4 consecutive sessions before t-HFS was applied. After session s4 (s4 = pre condition), HFS was applied. After t-HFS, discrimination thresholds were significantly reduced. This reduction persisted up to 24 hours after termination of t-HFS, indicating a long-lasting alteration in the individual percept. One week after t-HFS, tactile discrimination thresholds recovered to baseline conditions.

During the four initial training sessions (s1–s4 = pre) all subjects achieved a stable baseline of discrimination performance on their right d2 as estimated by repeated measures ANOVA with factor SESSION (F_(3,39) _= 0.759; p = 0.524; n = 14). After applying t-HFS for 20 minutes discrimination thresholds on the right d2 were reduced. Discrimination thresholds were 1.60 ± 0.08 mm before t-HFS and 1.34 ± 0.09 mm after stimulation resulting in an average gain of tactile performance of 0.25 ± 0.04 mm (rmANOVA pre vs. post: F_(1,13) _= 33.712; p < 0.0001; see Fig. 1 and 2). Linear correlation analysis (Pearson correlation coefficient) revealed no relation between baseline performance before stimulation was applied (pre-condition) and the individual gain in performance found after 20 min of t-HFS (r = -0.090; p = 0.761; n = 14), indicating that baseline performance is not a predictor for perceptual changes evoked by t-HFS. However, the results demonstrate that tactile discrimination performance can be improved by a short time period of tactile HFS. Analysis of the time course of the recovery of the t-HFS-induced changes demonstrated that the discrimination improvement did not recover to baseline conditions 24 hours after termination of stimulation indicating long-lasting alterations in the individual percept that outlasted the stimulation for 24 hours (rmANOVA pre vs. rec 24 h; F_(1,13) _= 5.771; p = 0.032). Re-testing one week after t-HFS revealed that discrimination thresholds recovered to conditions found prior to t-HFS (rmANOVA pre vs. 1 week; F_(1,13) _= 2.577; p = 0.132; see Fig. 2). Calculation of d'prime corroborated the improvement of discrimination performance by showing an increase after t-HFS from 2.14 ± 0.10 (pre) to 2.42 ± 0.09 (post). Values remained high 24 hours after stimulation (2.44 ± 0.09) but returned to baseline after 1 week (2.13 ± 0.10).

#### Spatial specificity of large-field t-HFS-induced changes

**Figure 3:**
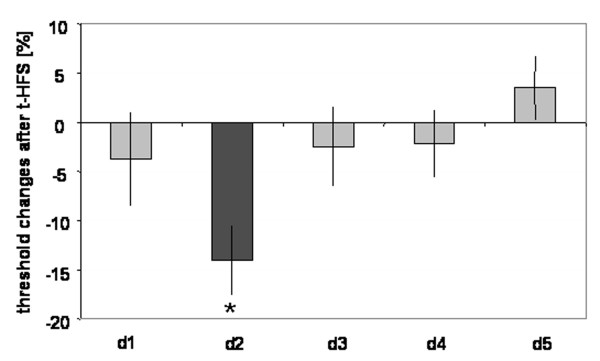
Spatial selectivity of t-HFS-induced effects. Percent changes in discrimination thresholds (n = 17) of all fingers of the right hand (d1–d5), while tactile HFS was applied to the right index finger (d2) for a period of 20 min. Apart from a significant lowering in discrimination thresholds on d2 after t-HFS, only little fluctuations could be observed for all other fingers. These findings demonstrate a lack of effects on others than the stimulated finger, which indicates a substantial local specificity of tactile HFS-induced changes.

In order to study the spatial selectivity of HFS-induced perceptual effects outside the stimulated d2, we looked for alterations in tactile performance on all other fingers of the right hand (see Fig. 3). Prior to t-HFS, post-hoc analysis corrected for multiple comparison (Bonferroni) revealed significant differences in discrimination thresholds for the middle-finger (d3) and ring-finger (d4) in comparison to d2 (post-hoc difference d3 vs. d2 p = 0.012; d4 vs. d2 p = 0.001). No statistical differences could be found between all other fingers.

Apart from a lowering in discrimination thresholds on d2 of 0.25 ± 0.05 mm (-14.00 ± 3.31%) after t-HFS (rmANOVA pre vs. post; F_(1,16) _= 13.506; p = 0.002), only little changes could be observed for all other fingers. We found alterations of thresholds of 0.07 ± 0.14 mm (-3.69 ± 9.70%) for the thumb (d1), 0.07 ± 0.17 mm (-2.43 ± 6.91%) for the middle-finger (d3), 0.07 ± 0.07 mm (-2.15 ± 2.63%) for the ring-finger (d4) and of -0.08 ± 0.09 mm (3.54 ± 3.30%) for the little-finger (d5). According to rmANOVA, none of these changes were significant. These findings demonstrate a clear absence of effects on other fingers than the stimulated d2, which indicates a substantial local specificity of the tactile HFS-induced changes (see Fig. 3).

#### Effect of small-field tactile HFS on discrimination thresholds of the right d2

**Figure 4:**
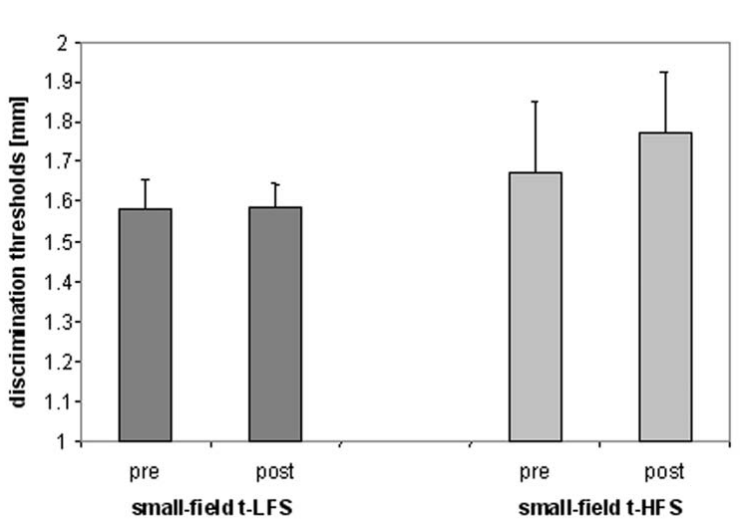
Effects of small field t-HFS and small field t-LFS on discrimination thresholds of the right d2.

Prior to small-field t-HFS, all subjects achieved a stable baseline performance (rmANOVA with factor SESSION: F_(3,12) _= 0.986; p = 0.432; n = 5). Stimulating the right index-finger (d2) using 20 minutes of small-field t-HFS resulted in no comparable changes of tactile discrimination thresholds (pre: 1.67 ± 0.17 mm; post: 1.77 ± 0.15 mm, rmANOVA pre vs. post: F_(1,4) _= 2.283; p = 0.205; n = 5, see Fig. 4).

### Tactile low-frequency stimulation (t-LFS)

#### Effect of large-field tactile LFS on discrimination thresholds of the right d2

**Figure 5:**
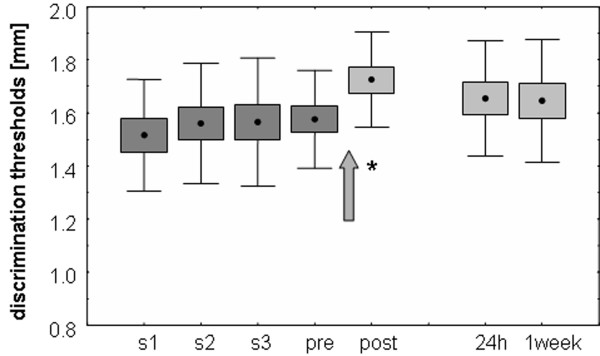
Psychophysical effect of large-field t-LFS on tactile discrimination thresholds of the right d2. Average data from all subjects of group 2 (n = 13). Dots represent mean thresholds, boxes show standard errors, and whiskers correspond to the standard deviation. Time of t-LFS application (20 minutes) on the right d2 is indicated by an arrow. Shown are the results from 4 consecutive sessions before LFS was applied. After session s4 (s4 = pre condition), t-LFS was applied. After t-LFS, discrimination thresholds were significantly increased, indicating impaired tactile performance. 24 hours after termination of t-LFS, discrimination thresholds recovered to baseline conditions. Reassessment of thresholds 1 week later revealed stable performance.

We assessed the outcome of large-field t-LFS on 2-point discrimination in 13 right-handed subjects. All subjects achieved a stable baseline performance, as estimated from repeated assessment of thresholds over 4 consecutive sessions (rmANOVA with factor SESSION F_(3,36) _= 0.052; p = 0.984, see Fig. 1 and 5). Under pre condition, discrimination thresholds were 1.57 ± 0.06 mm for the right d2. After 20 min of t-LFS, discrimination performance of the right d2 was impaired in all subjects as indicated by a significant increase in discrimination thresholds of 0.15 ± 0.04 mm, from 1.57 ± 0.06 mm to 1.72 ± 0.04 mm (rmANOVA with factor SESSION F_(1,12) _= 10.608; p = 0.007, see Fig. 5). Linear correlation analysis (Pearson correlation coefficient) revealed no significant relation between the individual performance before stimulation was applied (pre-condition) and the individual change in performance (r = 0.485; p = 0.093; n = 13). Analysis of the time course of stability of the effects revealed that discrimination thresholds recovered to baseline conditions 24 h after termination of t-LFS (rmANOVA with factor SESSION (pre vs. rec, n = 13) F_(1,12) _= 1.209; p = 0.293) implying that the t-LFS-induced impairment was less persistent than the improvement observed after t-HFS. Additional measurements one week after LFS application showed that discrimination thresholds remained unchanged as compared to baseline conditions (rmANOVA with factor SESSION (pre vs. rec 1 week, n = 13) F_(1,12) _= 0.958; p = 0.347, see Fig. 5). The decline of discrimination performance was confirmed by calculation of d'prime. We found a decrease after t-LFS from 2.29 ± 0.08 (pre) to 2.11 ± 0.08 (post). Values returned to baseline 24 hours after stimulation (2.27 ± 0.08) and remained stable after 1 week (2.32 ± 0.10).

#### Spatial specificity of large-field t-LFS-induced changes

**Figure 6:**
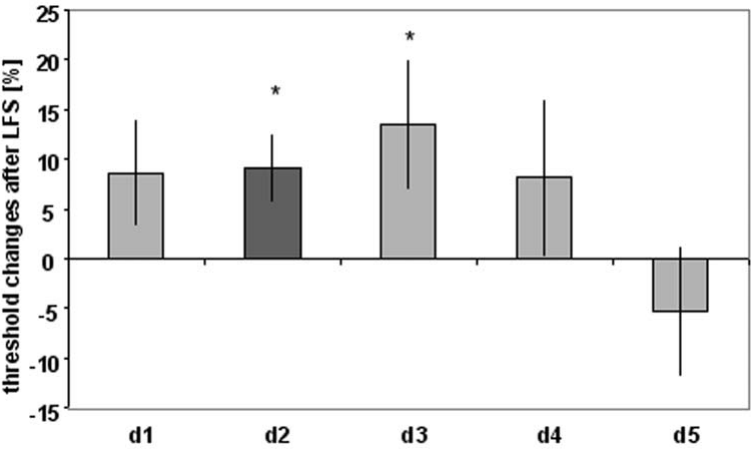
Spatial selectivity of t-LFS-induced effects. Percent changes in discrimination thresholds (n = 16) of group 4 of all fingers of the right hand (d1–d5), while tactile LFS was applied to the right index finger (d2) for a period of 20 min. In contrast to t-HFS, 20 minutes of t-LFS induced a significant decline in discrimination thresholds not only on d2, but also on d3 indicative for some transfer of plastic changes to other fingers.

Similar to the experiments described for t-HFS, we explored whether besides d2 the other fingers of the right hand were also affected by t-LFS. Baseline discrimination performance (d1–d5) on the right hand (n = 16) before t-LFS did not differ from the subpopulation that was used to study the local specificity of t-HFS-induced changes except for d3 (paired t-test: p = 0.032). In contrast to t-HFS, brief periods of t-LFS (20 min) resulted in changes of tactile discrimination thresholds not only on the right stimulated d2 (rmANOVA with factor SESSION (pre vs. post) F_(1,15) _= 6.985; p = 0.018), but also on the adjacent finger d3 (rmANOVA with factor session (pre vs. post F_(1,15) _= 6.963; p = 0.019, see Fig. 6). We found a change of thresholds of -0.12 ± 0.08 mm (8.64 ± 5.42%) for the thumb (d1), of -0.20 ± 0.07 mm (13.48 ± 6.48%) for the middle-finger (d3), of -0.14 ± 0.15 mm (8.17 ± 7.86%) for the ring-finger (d4) and of 0.17 ± 0.20 mm (-5.21 ± 6.62%) for the little-finger (d5). With the exception of d5, thresholds of the remaining fingers showed also some increase in thresholds, that were, however, not significant (see Fig. 6).

#### Effect of small-field t-LFS on discrimination thresholds of the right d2

Prior to small-field t-LFS, all subjects achieved a stable baseline performance (rmANOVA with factor SESSION: F_(3,12) _= 0.971; p = 0.438; n = 5). Moreover, baseline performance did not differ between the groups tested with either small-field t-LFS or small-field t-HFS (rmANOVA with factor GROUP: F_(1,8) _= 0.459; p = 0.517). Applying 20 minutes of small-field t-LFS resulted in no changes of tactile discrimination thresholds (pre: 1.58 ± 0.07 mm; post: 1.58 ± 0.06 mm, rmANOVA pre vs. post: F_(1,4) _= 0.03; p = 0.957, see Fig. 4).

## Discussion

The psychophysical results shown provide the first evidence for the effectiveness of t-HFS and t-LFS protocols to evoke tactile performance changes in human subjects. We found that brief periods of intermittent high-frequency tactile stimulation (t-HFS @ 20 Hz) applied to the skin of the right d2 induced a lowering of 2-point discrimination thresholds on that finger. Most interestingly, 24 hrs after t-HFS 2-point discrimination thresholds on the right d2 were still lowered in comparison to baseline indicating that brief periods of t-HFS can induce relatively long-lasting perceptual changes. These changes were spatially very specific as we did not observe any changes on the neighboring fingers. In contrast to t-HFS, 20 minutes of continuous low-frequency stimulation (t-LFS @ 1 Hz) led to a decline in tactile discrimination performance on the right stimulated d2. However, these changes were less lasting and recovered to baseline after 24 hours. T-LFS effects differed further in terms of spatial specificity. Impaired tactile performance was also observed on the right middle-finger. A central finding was that for both t-LFS and t-HFS perceptual changes were dependent on the size of the stimulated skin area. No changes at all were observed when the area was very limited (< 1 mm^2^) implying the need for spatial summation.

Peripheral tactile stimulation allows the systematic alteration of input statistics according to parameters such as stimulation frequency, duration, temporal structure and number of applied stimuli in order to study perceptual changes in the tactile domain [17]. For example, a peripheral tactile coactivation protocol as used in previous studies consisted of tactile stimuli that were presented at different interstimulus intervals from 100 to 3000 ms in pseudorandomized order with a mean stimulation frequency of 1 Hz to the tip of the index finger [7,18]. Testing 2-point discrimination thresholds before and after 3 hours of coactivation revealed an improvement in tactile discrimination [7]. Compared to the present study, a similar frequency specific dependency has been described for repetitive transcranial magnetic stimulation (rTMS) over SI. For example, brief periods of high-frequency rTMS applied over SI significantly improved 2-point discrimination thresholds [19,20] whereas 1 Hz rTMS impaired frequency discrimination [21].

In the current study stimulation protocols used to elicit LTP and LTD in vivo and in vitro [1,2,22] were adopted and applied in form of peripheral tactile stimulation. Several lines of evidence strongly suggest that improvement in 2-point discrimination evoked by peripheral stimulation reflects LTP-like plasticity in SI. First, the amount of improvement in 2-point discrimination correlates directly with the amount of map reorganisation in SI [8,9,23]. Secondly, its duration is in the order of 2–4 hours [7]. Thirdly, the improvement of 2-point discrimination can be abolished by antagonists at the N-methyl-D-aspartate receptor [11].

T-HFS and t-LFS differed from the standard coactivation protocol using irregular pulse trains with inter-pulse intervals between 100 and 3000 ms with respect to regularity, duration, number of applied stimuli, instantaneous frequency and temporal structure. In the present study, both t-HFS and t-LFS were applied for a time period of 20 min resulting in a total number of 4000 pulses in t-HFS and 1200 pulses in t-LFS. In contrast, in the standard coactivation protocol described above, a reduction of the stimulation period to 30 min with approximately 1800 tactile stimuli was not sufficient to drive plastic changes [7]. Based on these findings, it is conceivable that in addition to frequency the temporal structure, but not the duration of stimulation determine the outcome of tactile behavioral changes.

In the present experiments, both high- and low-frequency tactile stimulation were applied in two forms that differed according to the size of the skin area stimulated and the amplitude of probe movement. Because the amplitude of the small-field stimulation was higher than that of the large-field stimulation (200 vs. 100 microns), a larger effect on discrimination might have been expected, which was, however, not the case. Instead, the clear lack of effects evoked by small-field stimulation in both the t-HFS and t-LFS condition implies that certain spatial summation requirements need to be fulfilled in order to drive behavioral changes. Because small-field stimulation is clearly above threshold for evoking distinct sensation felt by the subjects, it is reasonable to assume that stimulation of this type also activates neurons located in the finger representation of somatosensory cortex. However, because of its limited size this cortical activation is not sufficient to overcome the threshold mechanisms to initiate plastic processes.

In all cases the protocols we here employed were local, restricting stimulation to the tip of the index finger, which was effective to alter 2-point discrimination on the skin regions stimulated. Therefore, another aim of the present study was to test whether tactile stimulation of the index finger also effects 2-point discrimination of the neighboring fingers. For t-HFS no changes could be detected on other than the stimulated finger which indicates a substantial spatial specificity with no transfer and spread on neighboring fingers. For t-LFS, the situation was quite different, as a significant impairment of threshold was not only observed for the stimulated index finger, but also for the middle finger. After t-LFS, thresholds were also higher for d1 and d4, although these changes were not significant (Fig. 5). Accordingly, the suppressive effects of t-LFS were less local with substantial spread across neighboring finger. Conceivably, locality and spread of plastic changes allow insight into connectivity pattern of cortical networks undergoing these changes. However, at present little is known how presumably LTP- or LTD-like processes have differential effects on the topography of cortical maps.

A number of studies have provided evidence according to which prolonged, but unattended stimulation is ineffective to drive plastic changes [24-26]. On the contrary, the observation that improvement in 2-point discrimination could be obtained using a passively applied stimulation protocol is in line with a recent study showing that perceptual changes occur even without awareness by repetitive exposure to stimuli that are below the threshold of visibility and that are irrelevant to the central task [27]. Their findings as well as ours described here show that tactile performance improvements can occur not only under training conditions, but also in situations of passive tactile stimulation. We suggest that the key to tactile performance improvements is to boost stimulus related responses that are normally insufficient to drive plastic changes [13]. Among these, factors such as attention and reinforcement might also play crucial roles.

The fact that in all cases under all conditions tested the discrimination thresholds on d5 remained unaffected can be taken as an argument against nonspecific side effects such as overstimulation, habituation, and alterations in local blood circulation or finger temperature. Influences of this type seem also very unlikely to play a role because of the opposite direction of effects evoked by using different stimulation frequencies. Also, systematic shifts in attention dependent of stimulation frequency would have likely impacted to similar extents the tactile performance of all fingers. According to recent unpublished data from our group, attending the coactivation stimuli during coactivation or being distracted from them had no effect on the overall outcome of coactivation. Therefore, the differential effects in 2-point discrimination induced by tactile high- and low-frequency stimulation are unlikely to be mediated by attentional processes. Interestingly, stimulation that effectively controls the timing of applied stimuli, as it is the case in peripheral tactile stimulation, might short cut the role of attention by producing the same conditions as attention, such as synchronous firing or increased probability of firing in specific temporal order among groups of neurons [28,29].

## Conclusion

In summary, the reported changes in 2-point discrimination in human subjects induced by either t-HFS or t-LFS provided first evidence for the perceptual relevance of stimulation protocols resembling those used in cellular LTP and LTD studies. Conceivably, further extension of these approaches will be beneficial for the understanding of possible mechanisms underlying tactile performance changes, and for developing novel strategies in neurorehabilitation.

## Methods

### Subjects

We tested a total number of 70 healthy subjects between 20 and 36 years (mean age 24.62 ± 4.42 years (SD)) in a psychophysical task using tactile spatial 2-point discrimination as a marker for alterations in human tactile perception. Subjects were randomly allocated to 2 experimental conditions (high-frequency tactile stimulation (t-HFS), n = 36; low-frequency tactile stimulation (t-LFS), n = 34): For an overview of subject's assignment to different tests see Table 1. According to the Oldfield questionnaire for the assessment of handedness [14], all subjects were right-handed. Subjects gave their written informed consent before participating. The protocol was approved by the local ethics committee of the Ruhr-University Bochum. The project protocol was performed in accordance with the 1964 Declaration of Helsinki.

**Table 1:** Summary of experimental groups

**Experimental Test Total number of subjects**	**Right d2**	**Right d1–d5**
Large-field t-HFS (n = 31)	n = 14	n = 17
Small-field t-HFS (n = 5)	n = 5	n.a.
Large-field t-LFS (n = 29)	n = 13	n = 16
Small-field t-LFS (n = 5)	n = 5	n.a.

### 2-point discrimination

Tactile 2-point discrimination on the fingers was assessed using the method of constant stimuli as described previously (for an overview see [7,11,15]). To overcome problems in the use of 2-point measurements associated with hand held probes, we used a specifically designed apparatus that allows a standardized and objective form of testing. In brief, seven pairs of rounded needle-probes (diameter 200 μm) with separation distances between 0.7 to 2.5 mm in 0.3 mm steps were used. For control, zero distance was tested with only a single needle-probe. The number of single-needle presentations was 1/8, i.e. 8 presentations in one session. The probes were mounted on a rotatable disc that allowed switching rapidly between distances. To accomplish a rather uniform and standardized type of stimulation the disc was installed in front of a plate that was movable up and down. The arm and fingers of the subjects were fixated on the plate and the subjects were then asked to move the arm down. The down-movement was arrested by a stopper at a fixed position above the probes. The test finger was held in a hollow containing a small hole (diameter 15 mm) through which the distal phalanx of the finger came to touch the probes approximately at the same indentations in each trial. The probes were always presented parallel the fingertip. Each distance was presented 8 times in randomized order resulting in 64 single trials per session. Subjects were aware that there are single needle-probes presented but not how often. The subjects had to decide immediately after touching the probes if he or she had the sensation of one or two tips by answering "one" or two". After each session individual discrimination thresholds were calculated. The summed subject's responses ("1" for one tip and "2" for two tips) were plotted against the tip distance as a psychometric function and were fitted with a logistic regression method (SPSS version 10.01). Thresholds as a marker for individual tactile performance were taken at that point at which 50% correct responses were reached. To provide evidence that a change in discrimination sensitivity is unlikely to be due to changes in the response criterion, we calculated the false alarm as well as the hit rates and the discrimination index (d' value) for the subject groups tested with t-HFS and t-LFS on d2 [16]. The d' value equals the difference between the z-transform of the hit rate (z (H)) and the z-transform of the false alarm rate (z (F)) (d' = Z (H) – z (F)). The hit rate describes the probability of discriminating two tips whenever two tips are presented, whereas the false alarm rate describes the probability of detecting two tips when only one is present. In order to carry out the numerical calculation in case of zero false alarm rates, the false alarm rate was set to 0.01.

### Stimulation protocols

#### Tactile high-frequency (t-HFS) and low-frequency (t-LFS) stimulation protocols

t-HFS and t-LFS consisted of brief rectangular pulses (10 ms duration) of tactile stimuli that were applied to the distal phalanx of the right index-finger (d2) to the position where 2-point discrimination was performed. Pulse trains required to drive the stimulators were stored digitally as TTL pulses and played back via MP3 player allowing unrestricted mobility of the subjects during the stimulation period. For t-HFS, stimulation trains consisted of 20 single pulses within 1 s with an inter-train interval of 5 s. T-LFS was applied continuously at a stimulation frequency of 1 Hz. Duration of t-HFS or t-LFS application was 20 min, resulting in a total number of 4000 pulses for t-HFS and 1200 pulses for t-LFS. In all cases, subjects were instructed not to attend the stimulation but to resume their daily routine. Although the spectral power of the single pulses was "high-frequency" (pulse duration 10 ms), we use the term "low" or "high-frequency" stimulation to describe the number of pulse per second (pps) in terms of Hz to connect to the terms used in cellular studies of brain plasticity.

For both t-HFS and t-LFS we used two variants that differed with respect to the skin-size stimulated. In the standard protocol a skin area of approximately 50 mm^2 ^on the tip of the IF was stimulated. We called this protocol large-field stimulation. In the other version a very restricted (point-like) skin area of only 0.8 mm^2 ^was stimulated, which is termed small-field stimulation. For application of the large-field stimulation, we used a device consisting of a solenoid connected to a flexible membrane with a diameter of 8 mm, which was fixed to the tip of d2. Laser vibrometer measurements revealed that the actual amplitude of the membrane movement was about 100 microns. For application of the small-field stimulation, we used a tiny stimulator consisting of a small needle probe (diameter 0.5 mm) that was also taped to the tip of the right d2. The probe was connected to the switch of a relay that changed its vertical position whenever a voltage of 5 V was applied. Changes in voltage were controlled via a small electrical amplifier that was driven by the stimulation pulses from the MP3 player. Amplitude of the probe movement was in the range of 200 microns. By comparing the effects induced by either large- or small field stimulation information about spatial summations processes can be obtained. Sensations elicited by the stimulation were very soft but perceptible resembling a very light touch on the stimulated skin site. In order to study the spatial selectivity of t-HFS/t-LFS-induced perceptual effects outside the stimulated d2, we studied alterations in tactile performance on all other fingers of the right hand in two additional groups (Table 1).

#### Experimental Schedule

**Figure 7:**
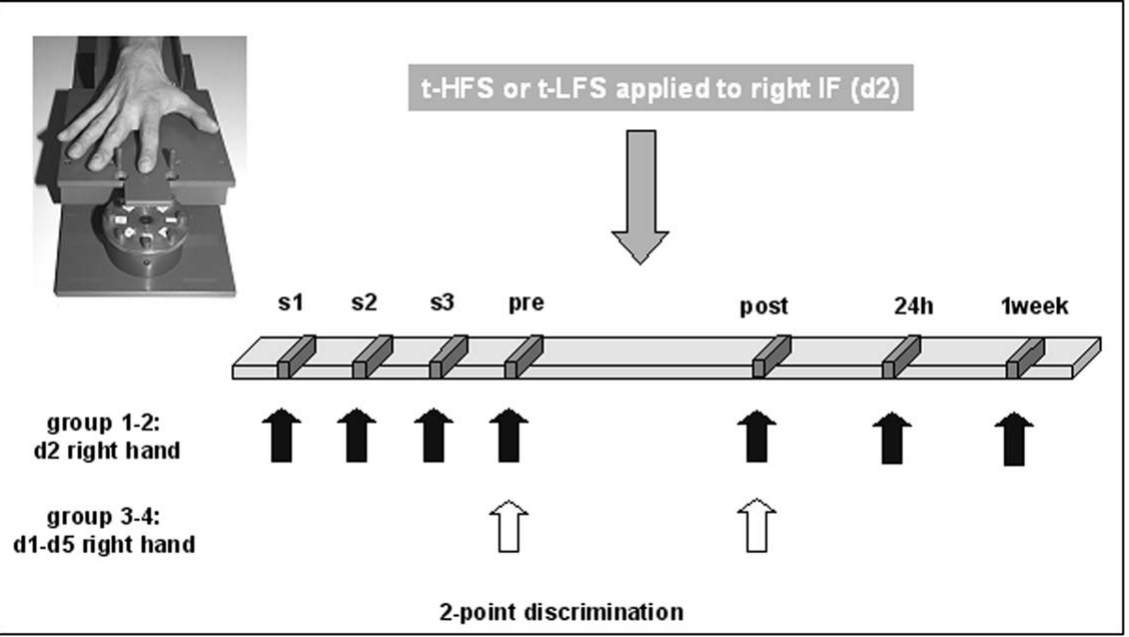
t-HFS/t-LFS schedule and procedure. Session 1–4 (s4 = pre-condition) served to create a stable baseline performance. After session 4 (pre-condition), either t-HFS (group 1, n = 14) or t-LFS (group 2, n = 13) was applied to the right index-finger (d2) for a duration of 20 minutes. After termination of t-HFS/t-LFS post measurements of tactile discrimination thresholds were performed for the right d2. Additionally, 24 hours (rec-24 h as well as one week (rec-1 week) after t-HFS/t-LFS the measurement of 2-point discrimination thresholds was repeated. For group 3 (t-HFS; n = 17) and group 4 (t-LFS; n = 16), which both address the question of the spatial selectiviy of the induced effects, tactile discrimination thresholds for all fingers of the right hand (d1–d5) were assessed before (pre) and after (post) t-HFS/t-LFS.

The experiment consisted of two different components: (1) the measurement of 2-point discrimination thresholds; (2) tactile HFS or LFS of 20 minutes duration each to induce perceptual changes on the right d2. Prior to t-HFS/t-LFS, discrimination thresholds were tested on 4 consecutive sessions (session (s) 1–4) on one day in order to obtain a stable baseline performance. Testing 2-point discrimination for each session lasted for approx. 5 minutes and was separated by 2 minutes (Fig. 7). In experiments that addressed the spatial selectivity, all fingers (d1–d5) of the right hand were additionally tested in session s4 (pre). Previous studies had shown that the initial task familiarization completely generalizes to the other fingers [7-9,11]. For all experimental groups, thresholds derived during session 4 (s4) were taken as "baseline" or "pre-condition", and were used for further analysis. After s4 (pre-condition), either t-HFS or t-LFS was applied for 20 minutes. Re-assessment of tactile performance on d2 or on d1–d5 was repeated approximately 10 minutes after termination of t-HFS/LFS in session 5 (post-condition) to investigate in how far stimulation protocols known to induce LTP and LTD in cellular studies also have a behavioral relevance on tactile performance in the intact human brain. Tactile performance was retested on the right d2 24 hours after termination of t-HFS and t-LFS as well as one week later to study the time course of stability and reversibility of t-HFS- and t-LFS-induced behavioral changes (see Fig. 7 for experimental design).

### Data analysis

Psychophysical data were statistically analyzed using repeated measures ANOVA (rmANOVA) with factor SESSION or univariate ANOVA (uANOVA) with factor FINGER or GROUP as well as Student's paired t-tests, post-hoc analysis corrected for multiple comparisons (Bonferroni) and correlation analysis (Pearson correlation coefficient). Data were normally distributed as evaluated by Kolmogorov-Smirnov tests. Inspection of the data showed that false alarms were zero under each condition. All data are expressed as mean ± s.e.m. (standard error of mean).

## Authors' contributions

PR conceived of the study, carried out parts of the psychophysical measurements, performed the statistical analyses, and drafted the manuscript. BB and SF carried out parts of the psychophysical experiments. BB also helped to draft the manuscript. TK participated in the study design. HRD participated in the study design and coordinated and helped to draft the manuscript. All authors read and approved the final manuscript.
